# The Role of the Val66Met Polymorphism of the Brain Derived Neurotrophic Factor Gene in Coping Strategies Relevant to Depressive Symptoms

**DOI:** 10.1371/journal.pone.0065547

**Published:** 2013-06-18

**Authors:** Warren Caldwell, Opal A. McInnis, Robyn J. McQuaid, Gele Liu, John D. Stead, Hymie Anisman, Shawn Hayley

**Affiliations:** Department of Neuroscience, Carleton University, Ottawa, Ontario, Canada; Chiba University Center for Forensic Mental Health, Japan

## Abstract

Disturbances of brain derived neurotrophic factor (BDNF) signalling have been implicated in the evolution of depression, which likely arises, in part, as a result of diminished synaptic plasticity. Predictably, given stressor involvement in depression, BDNF is affected by recent stressors as well as stressors such as neglect experienced in early life. The effects of early life maltreatment in altering BDNF signalling may be particularly apparent among those individuals with specific BDNF polymorphisms. We examined whether polymorphisms of the Val66Met genotype might be influential in moderating how early-life events play out with respect to later coping styles, cognitive flexibility and depressive features. Among male and female undergraduate students (N = 124), childhood neglect was highly related to subsequent depressive symptoms. This outcome was moderated by the BDNF polymorphism in the sense that depressive symptoms appeared higher in Met carriers who reported low levels of neglect than in those with the Val/Val allele. However, under conditions of high neglect depressive symptoms only increased in the Val/Val individuals. In effect, the Met polymorphism was associated with depressive features, but did not interact with early life neglect in predicting later depressive features. It was further observed that among the Val/Val individuals, the relationship between neglect and depression was mediated by emotion-focused styles and diminished perceived control, whereas this mediation was not apparent in Met carriers. In contrast to the more typical view regarding this polymorphism, the data are consistent with the perspective that in the presence of synaptic plasticity presumably associated with the Val/Val genotype, neglect allows for the emergence of specific appraisal and coping styles, which are tied to depression. In the case of the reduced degree of neuroplasticity expected in the Met carriers, early life adverse experiences are not tied to coping styles, and hence less likely to be translated into depressive states.

## Introduction

Increasing attention has been devoted to the possibility that growth factors, such as brain derived neurotrophic factor (BDNF), through actions on neurogenesis and neuroplasticity, contribute to the evolution of depressive disorders [1]. In this regard, BDNF, which normally provides trophic support for neurons, is reduced in response to stressors [2], and serum levels of this trophic factor were lower in depressed patients than in controls [3], [4]. In line with a role for BDNF in depression, low serum BDNF levels were accompanied by a poorer treatment response than among those depressed individuals with higher BDNF levels [5]. However, positive treatment responses elicited by SSRI treatment were accompanied by increased serum BDNF levels [6], [7]. Although these studies indicate that BDNF might be a biomarker for depression, they don’t speak to the possibility that peripheral BDNF might actually contribute to this disorder. This said, in animals, systemic BDNF treatment reduced signs of anxiety in several behavioral tests coincident with increased hippocampal neurogenesis, suggesting a causal role for peripheral BDNF in promoting behavioral changes [8].

Several polymorphisms (e.g., 5-HTTLPR) have been described that are associated with elevated severity or frequency of depressive disorders or are associated with depression provided that individuals have also encountered either early life stressors or major life challenges [9]–[11]. Likewise, a polymorphism was identified wherein a valine (Val) to methionine (Met) substitution occurred in the 5' pro-region of the human BDNF protein [12]. In mice with a single nucleotide polymorphism of the BDNF gene, BDNF protein levels were reduced and survival of newly born subventricular zone neurons were impaired [13], and selectively knocking out the gene for BDNF attenuated the effects of antidepressant medication in a forced swim test [14]. Further, the depressive-like behaviors associated with stressor treatments in mice that were Met carriers could be reversed by desipramine, but not fluoxetine, indicating class selectivity regarding the actions of antidepressant agents [15]. Studies in humans have provided less consistent results, and a meta-analysis revealed a better response to antidepressant treatment among Val66Met heterozygous individuals than in Val/Val depressed patients, particularly in Asian populations [16]. This said, it was also reported that the Val66Met mutation was accompanied by non-responsiveness to antidepressant treatment [17].

There have been reports indicating that BDNF signaling is not only affected by recent stressors encountered, but also by stressors experienced in early life [18], and it appeared that the effects of early life maltreatment in reducing BDNF levels was particularly pronounced in Met carriers [19]. Moreover, negative affectivity was associated with stressors encountered during childhood, an outcome that was most notable among Met carriers [20], [21]. In contrast to these findings, however, BDNF was less affected by early adversity in Met carriers, even though the Met allele was associated with increased depression susceptibility [22]. Further, it was indicated that the BDNF Met allele may actually have a protective effect among individuals who carried the SS allele for 5-HTTLPR and who had experienced childhood abuse [23].

It has been known for some time that the case for BDNF involvement in depression has not been consistent [24]. It has been suggested that it might be propitious to consider that genes, such as those related to 5-HTTLPR and Val66Met should not be considered to be ‘vulnerability factors”, but instead viewed as ‘plasticity factors” that favor depression provided that particular environmental challenges had been encountered [25], [26]. It could be argued that individuals with the Val-Val allele might gain the most from positive nurturing environments, but because of their elevated plasticity they might also be most vulnerable to the adverse effects of a poor early life environment. Conversely, in those with the Met allele, the diminished plasticity might be endogenously more prone to become depressed, but the limited plasticity would also be less likely to result in positive or negative early life experiences affecting behavioral or cognitive outcomes.

To a significant extent, the way individuals appraise and cope with stressors are linked to depressive disorders. Individuals with high levels of negativity or who favor emotion focused coping generally exhibit greater or more frequent depression than do individuals with a problem-focused coping orientation [27]–[30]. Likewise, depressed individuals are less flexible in the coping strategies used, whereas those individuals who are flexible and able to move from one coping strategy to another as situations demand are less likely to be depressed [31], [32]. This doesn’t necessarily suggest that emotion-focused coping is necessarily maladaptive [33], [34], nor does it imply that coping styles are etiological significant in the development of depression, even though certain emotion focused methods, such as rumination, were found to be predictive of later depression [27], [35], [36]. However, it was reported that Met carriers are more likely to use rumination as a coping style [37], [38].

How early life events might come to increase later vulnerability to depressive disorders has been a question of intense interest and analysis. It is possible that negative early experiences set a developmental trajectory so that allostatic overload might be more likely to occur, hence leading to pathology [39]. For instance, this outcome could occur because negative early events alter the way in which individuals appraise and cope with stressors [40], [41]. It is also possible that in the presence of genes that affect plasticity, early events might have particularly marked repercussions. Of course, these views are not exclusive of one another, but might act additively or synergistically. In light of these perspectives, in the present investigation we assessed whether early life adversity in the form of self-perceived neglect was associated with depressive symptoms, and whether this relationship was mediated by particular coping styles and dimensions of cognitive flexibility. As well, we determined whether the Val66Met genotype, and by extension BDNF levels, might be influential in moderating how early events play out with respect to later coping, flexible thinking and depressive features.

## Methods

### Participants

Participants included 124 Carleton University first and second year students (82 females and 42 males), with a mean age of 21.97 (*SD* = 5.69) who had been recruited through the university’s online computerized recruitment system. Self-reported ethnicity included White (53.2%, *n* = 66), Arab (13.7%, *n* = 17), Black (12.9%, *n* = 16), South Asian (7.3%, *n* = 9), Asian (6.5%, *n* = 8), South East Asian (1.6%, *n* = 2), Latin American (1.6%, *n* = 2), Aboriginal (0.8%, *n* = 1), and other (e.g., mixed ethnicity, 2.4%, *n* = 3). A diagnosis of a current mental disorder was recorded among 7 individual (depression *n* = 4, bipolar disorder *n* = 1, generalized anxiety disorder *n* = 1, and post traumatic stress disorder *n* = 1). Of those with a mental health disorder, all were female with a mean age of 24.75 years. Four of the seven participants reported currently being treated with psychoactive drugs. Of those who reported medication use, two individuals were taking paroxetine, one was taking citalopram and another was taking sertraline.

### Procedure

Once signed informed consent was obtained, participants responded to a series of demographic questions as well as measures of current depressive symptoms, childhood maltreatment, coping-style and cognitive flexibility in response to stressors. Saliva samples for DNA genotyping were taken following the demographic questions. Upon completion of the study, all participants were debriefed and compensated with course credit. This session took up to 1.5 hours to complete. All procedures in the current study were approved by the Carleton University Ethics Committee for Psychological Research. Signed consent was obtained for all participant and no minors (participants under the age of 18) were used in the study.

### Genotyping

Samples for genotyping were collected using Oragene OG-500 collection kits (DNA Genotek, Inc., Ottawa, Ontario, Canada). Genomic DNA was extracted from the sample collection kit according to the manufacturer’s instructions and diluted to approximately equal concentration (50 ng/µL). Genotyping was conducted using polymerase chain reaction (PCR), followed by the restriction fragment length polymorphism procedure. PCR conditions were 94°C for 3 minutes, then 32 cycles of 94°C for 40 seconds, 68°C for 30 seconds, and 72°C for 45 seconds, and then 70°C for 5 minutes. Amplification reactions were performed in a total volume of 15 µl, containing approximately 1 µL (50 ng) of genomic template, 0.4 µL of each primer (concentration 10 µM), 1.2 µL of dNTP, 1.5 µL 10X Buffer, 0.6 µL of MgCl_2_, 0.21 µL of Taq polymerase, and 10.69 µL of water. The PCR product was digested using the restriction enzyme Hsp92II and an aliquot of the PCR product was saved in its undigested state. The enzyme was selected based on a previous study demonstrating its ability to differentiate the val66met polymorphism [42]. The digestion reaction was performed in a total volume of 20 µL containing 5 µL of PCR product, 2 µL of 10X Buffer, 0.2 µL of BSA, 0.75 µL of Hsp92II, and 12.05 µL of water. Hsp92II digests the Val polymorphism into two fragment lengths (20 bp and 277 bp) and the Met polymorphism into three fragment lengths (20 bp, 77 bp, and 200 bp). Subsequently, digested and undigested PCR products were electrophoresed on 2% agarose gel and then visualized.

The allele distribution of the Val66Met polymorphism was 90 val/val individuals (33 male, 57 female) and 31 Val/Met and Met/Met individuals (7 male, 24 female). Due to the relative infrequency of the Met/Met gene variant (n = 3), the data from both the Val/Met and Met/Met genotypes were grouped for analyses. Three individuals were excluded from analyses because we were unable to determine a genotype from the sample they provided. No significant differences were found between the genotype groups based on age, F (1, 119) = 0.006, *ns*, sex, χ^2^ (1) = 2.067, *p* = 0.15, race, χ^2^ (8) = 10.842, *ns*, relationship status, χ^2^ (4) = 5.324, *ns*, or past mental disorder, χ^2^ (1) = 0.307, *ns*. Furthermore, distribution of current mental disorders across genotype was relatively even (Val/Val *n* = 4, Val/Met & Met/Met *n* = 3).

### Measures

#### Depressive symptoms

The 21- item Beck Depression inventory (BDI) [43] was used to assess depressive symptoms. For each item participants responded to one of four options which ranged from low to high depression symptomatology. Total scores were calculated by summing across all items (*α* = .90).

#### Coping-style

The 50- item Survey of Coping Profile Endorsement [29] assessed the means individuals use to cope. On a scale of 0 “never” to 4 “almost always”, participants indicated the extent to which they would use this as a way of dealing with problems or stressors in recent weeks. The factors to examine broad differences in coping were determined based on previous use of the SCOPE [29], and confirmed by a principal component analysis. Items were included on a factor when loadings were greater than 0.40. Emotion focused coping comprised of avoidance, rumination, emotional expression, other blame, passive resignation, and wishful thinking (α = 0.86); and problem focused coping comprised of problem solving, cognitive restructuring, active distraction, humor, and social support (α = 0.86).

#### Childhood maltreatment

The 31- item Childhood Maltreatment Questionnaire (short form) [44] assessed levels of maltreatment comprising psychological (*α* = .94), physical (*α* = .86), and sexual abuse (*α* = .89) as well as neglect (*α* = .67). Each item can be rated from 1 (never) to 5 (very often) indicating the frequency of experiences.

#### Cognitive Flexibility

The 20-item Cognitive Flexibility Inventory [45] measured flexibility in the face of stressful situations. Each statement was rated by the participant from 1 (Strongly disagree) to 7 (Strongly agree). Two dimensions of flexibility were assessed; awareness of multiple alternative causes and solutions to stressful scenarios as well as perception of being flexible with respect to control over stressful scenarios (i.e., having options available in order to control events and outcomes). Higher scores represent greater flexibility (*α* = .92) and greater perception of control (*α* = .87).

### Statistical Analyses

The statistical analyses were performed using SPSS for Windows 18.0 (SPSS Science, Chicago, Illinois, USA). Statistical significance was determined at p<0.05 (two-tailed). The N was sufficient to detect a small effect size at a statistical power level of 0.80 and a probability level of 0.05. Analyses assessing differences on depression scores, childhood maltreatment subscales, coping styles and dimensions of cognitive flexibility were assessed using univariate analysis of variance (ANOVAs) with a Bonferonni correction. Correlational analysis was performed using Pearson product moment correlations. Moderations were analyzed using hierarchical linear regressions, and the significant moderations were followed up using a web utility for simple slopes [46]. Moderated mediation analyses were conducted using bootstrapping procedures and confidence intervals based on 5000 resamples [47]. In all regression analyses standardized scores were used.

## Results

The depression scores among individuals in the Val/Val (M = 8.32; *S.E.* = 0.86) and the combined Val/Met and Met/Met (M = 9.77; *S.E.* = 1.48) conditions did not differ from one another, *F* (1, 119) = 0.72, *ns*. Similarly responses to the childhood maltreatment subscales did not differ based on genotype: physical, F (1, 119) = 0.03, *ns*, psychological, F (1, 119) = 0.75, *ns*, and sexual abuse, *F* (1, 119) = 1.01, *ns* or that of neglect, F (1, 119) = 0.01, *ns*. Females were found to endorse emotion focused coping more frequently than did males, *F* (1, 122) = 18.29, *p*<0.001 *η^2^* = 0.13, and they also reported less perceived control, *F* (1, 122) = 9.11, *p*<0.01 *η^2^* = 0.07. There were no Gender x Gene interactions in relation to either depression, coping or perceived control.

Examination of the relationship between childhood maltreatment and depressive symptoms revealed that psychological abuse and neglect were associated with depression scores, *r* = 0.30, *p*<0.01, and *r* = 0.26, *p*<0.01 respectively. Physical abuse was not related to later depression, and there were too few participants who reported sexual abuse (N = 5) to provide meaningful results. Thus, the possible moderating effect of genotype was only explored regarding the relationship between childhood psychological abuse and neglect with symptoms of depression.

**Figure 1:**
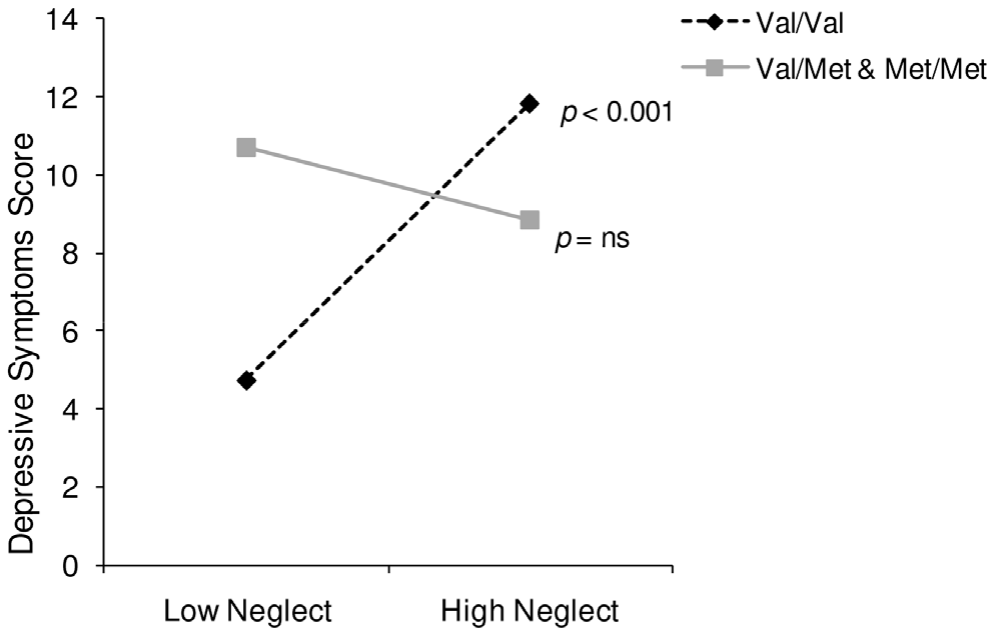
The relation between childhood neglect and depression score was moderated by BDNF genotype. Based on a hierarchical regression, childhood neglect interacted with BDNF genotype (Val/Val versus Val/Met & Met/Met) to predict depressive symptoms score. Among individuals with the Val/Val genotype, depressive symptoms were significantly increased at high levels of childhood neglect, whereas this relationship was not found for individuals with one or more Met allele.

A hierarchical linear regression was conducted to examine possible interactions between the childhood maltreatment subscales that were associated with depression and BDNF genotype. Genotype and psychological abuse were entered on the first step, and the Genotype x Psychological abuse interaction terms were entered on the second step. The moderating role of BDNF genotype between psychological abuse and depression was not significant, *b* = −1.07, *t* = −0.65, *ns*. Next a hierarchical linear regression was conducted to examine the interactive effects of neglect and genotype. In this regard, a significant Genotype x Neglect interaction predicted depression scores, *b* = −4.45, *t* = −2.95, *p*<0.01. The interaction was followed up with a simple slopes analysis [46]. As depicted in Figure 1, for individuals of the Val/Val BDNF genotype, depressive symptoms were significantly increased at high levels of childhood neglect (*p*<0.001). However, among individuals with one or more met allele, who showed relatively high levels of depression at low levels of neglect, a further increase of depression scores was not observed in the presence of high levels of neglect (*p* = 0.47).

**Figure 2:**
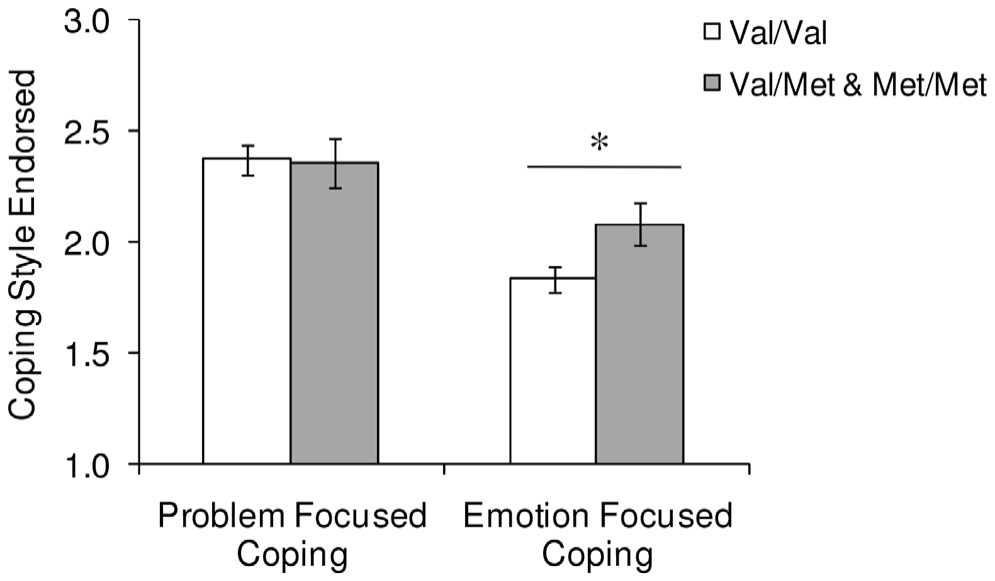
Coping style endorsement (mean±S.E.M.) of as a function of genotype. There were no differences in levels of problem focused coping styles endorsed among the BDNF genotypes (Val/Val versus Val/Met & Met/Met). However, endorsement of emotion focused coping styles differed among genotypes, such that Met allele carriers reported higher levels of emotion focused coping compared to Val/Val individuals. * *p*<0.05.

To examine potential pathways through which childhood neglect may predict depressive symptoms, the contribution of coping styles (i.e. problem and emotion focused) were examined. First, coping styles were examined to determine if levels varied among individuals of different BDNF genotype. Problem focused coping did not differ based on BDNF genotype *F* (1, 119) = 0.01, *ns.* However, emotion focused coping varied as a function of BDNF genotype, such that individuals with at least one Met allele endorsed more emotion focused coping than those with a Val/Val genotype, *F* (1, 119) = 4.70, *p*<0.05, *η^2^* = 0.04 (Figure 2).

**Figure 3:**
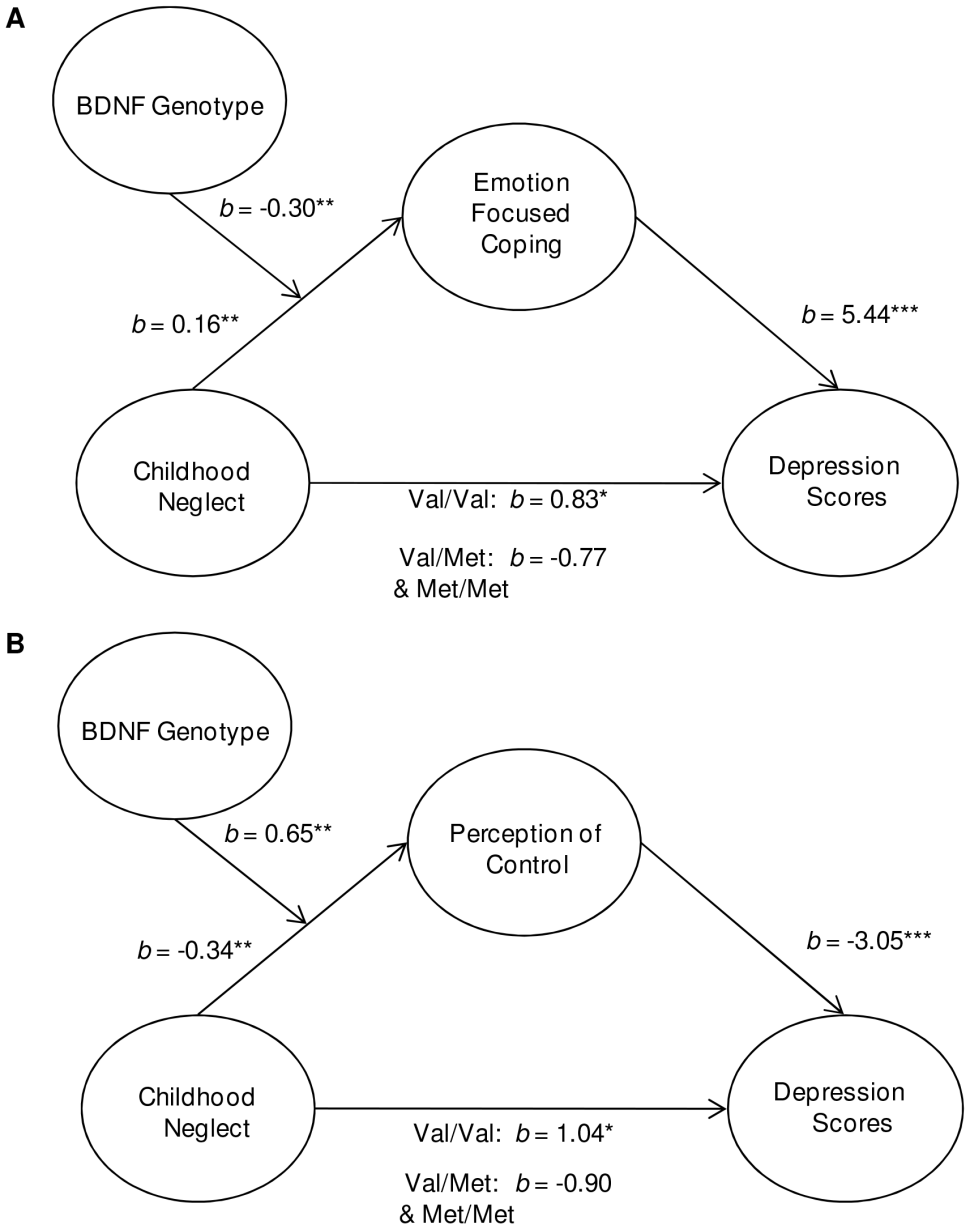
Schematic representations of the moderated mediation models. The relation between childhood neglect and depression scores through emotion focused coping was moderated by BDNF genotype (Val/Val versus Val/Met & Met/Met). This mediation model was found only to be significant among Val/Val carriers (A). Additionally, perceptions of control mediated the relationship between childhood neglect and depression scores, and once again this was only observed among those homozygous for the Val allele (B). *p<0.05, **p<0.01 and ***p<0.001.

As expected, problem focused coping was negatively related to depression scores, whereas emotion focused coping was positively associated with depressive symptoms, *r* = −0.31, *p*<0.01, and *r* = 0.43, *p*<0.001 respectively. The mediating role of coping styles in the relation between childhood neglect (predictor) and depression scores (outcome) was examined along with whether these relationships were moderated by BDNF genotype. Moderated mediation analyses were conducted using bootstrapping procedures and confidence intervals based on 5000 resamples [47]. The mediating role of problem focused coping in the relation between neglect and depression scores was not moderated by BDNF genotype. Interestingly, BDNF genotype did moderate the mediating role of emotion focused coping in the relation between neglect and depression scores (Figure 3A). More specifically, the relation between neglect and depression scores was mediated by emotion focused coping, but this mediation model was only significant for Val/Val carriers. Additionally, alternative models in which BDNF genotype moderates the path between coping and depressive scores were not significant, suggesting the importance of the interaction between neglect and BDNF genotype.

Another important pathway through which neglect might predict depressive symptoms is the extent to which individuals endorse cognitive flexibility when faced with stressful situations. Dimensions of cognitive flexibility were examined to determine if levels varied among individuals of different BDNF genotypes. It was determined that there were no differences between genotypes on measures of cognitive flexibility for consideration of alternatives, *F* (1, 119) = 1.00, *p* = 0.32, or perceived control when faced with stressors, *F* (1, 119) = 1.47, *p* = 0.23. Likewise, depression scores were not related to consideration of alternative solutions for stressful scenarios, *r* = −.16, *p* = 0.07. However, perceived control over stressful scenarios was strongly related to depression scores, *r* = −.50, *p*<0.001.

Thus, to further examine perceived control, only the control dimension was pursued. The mediating role of perception of control between childhood neglect (predictor) and depressive symptoms (outcome) was examined along with whether this mediation was moderated by BDNF genotype. Again, moderated mediation analyses were conducted using bootstrapping procedures and confidence intervals based on 5000 resamples [47]. Interestingly, the BDNF genotype was found to moderate the mediating role of perception of control between neglect and depression scores (Figure 3B). In this regard, perceptions of control mediated the relationship between childhood neglect and depression scores among individuals with the Val/Val genotype, but this was not observed among Val/Met & Met/Met carriers. Alternative models in which BDNF genotype moderated the path between perceived control and depressive scores were not significant.

## Discussion

Both depression and the Val66Met polymorphism have been associated with deficits in neurotrophic support [12], [48], [49], reduced size and function of the hippocampus and prefrontal cortex [50]–[53], as well as use of a ruminative coping style [37], [38], although the Val66Met polymorphism was not necessarily linked to the appearance of depression [54]–[56]. Indeed, as indicated earlier, the findings concerning the relationship between BDNF polymorphisms and depression have been inconsistent, and the suggestion had been made that the link between BDNF and psychopathology ought to be re-evaluated [24]. This said, there does seem to be reason to suppose that the BDNF mutation may contribute to depression in certain populations, including the elderly [57], [58] as well as individuals that might have encountered early life stressors, particularly those that entailed neglect.

As typically observed [29], [59] depressive symptoms in the present investigation were accompanied by increased emotion-focused and avoidant coping styles, coupled with reduced problem focused coping. Moreover, consistent with reports that emotion-focused responses, such as rumination, were more prominent in Met carriers [38], in the present study these individuals reported significantly greater endorsement of emotion-focused coping strategies (but not problem focused coping) than the Val/Val participants. These findings are clearly in line with the perspective that neurotrophic disturbances might contribute to appraisals or coping with stressful experiences [38] and might thus favor the development of depressive symptoms, although it is possible that the presence of depression encouraged the emotion-focused coping strategies.

It is particularly interesting, as seen in Figure 1, that depressive symptoms appeared elevated among Met carriers under ordinary conditions (i.e., in the absence of early life abuse). However, whereas high levels of self-reported early life neglect were associated with elevated depressive symptoms among Val/Val individuals, this relationship was not found among Met carriers. Moreover, it appeared that the relationship between childhood neglect and depressive features was mediated by elevated emotion-focused coping and diminished perceptions of control. However, once again, these mediated effects were prominent among the Val/Val individuals, but not among Met carriers. Rather, among Met carriers, the relation between childhood neglect and depression occurred irrespective of coping style and perceived control.

These findings, at first blush, seem contrary to the perspective that the early adverse events would have the most negative effects in the presence of the Met mutation and presumably diminished BDNF levels. Indeed, the present findings are contrary to those of other investigators who demonstrated augmented effects of early life stressors in Met carriers, including reduced hippocampal and prefrontal cortex volume, elevated depression and anxiety, neurotic personality traits, and increased heart rate reactivity to a startle stimulus [60]–[62]. Why the present findings regarding the interaction between early life stress and the BDNF polymorphism in relation to depression differed from earlier studies isn’t certain, especially given the similarity of the procedures that were used [61]. Yet, the present findings seem to follow from the perspective that Val/Val individuals, with adequate BDNF, would benefit most from a positive environment. However, in the presence of a negative environment, such as early life neglect, the adequate plasticity might contribute to how individuals subsequently cope with stressors, which could then influence vulnerability to stressor-related depression. Among Met carriers, basal levels of depression would be expected to be elevated, but owing to limited plasticity, neither positive nor negative early life events would further affect coping to the same extent.

Given previous studies linking the Val66Met polymorphism to hippocampal functioning [63] it was expected that the measures of cognitive flexibility would vary with this polymorphism. Although cognitive flexibility itself did not act as a mediator for the link between neglect and depressive symptoms, perceived control, a dimension of flexibility, served as a mediator in this regard, but once more this only occurred among those with the Val/Val genotypes. As discussed in relation to coping processes, this outcome seems to be consistent with the view that the Val/Val genotype is associated with greater plasticity compared to individuals with one or more Met allele. Thus among Val/Val individuals perceived control is diminished following neglect, which in turn, was linked to depression.

There are several limitations regarding the present findings. Foremost among these is that early-life stress was determined on the basis of retrospective self-reports that might be biased by the individuals’ current affective state, and indeed individuals might be unaware of events that occurred so many years earlier. Furthermore, the extent of the early abuse/neglect reported by most participants was relatively limited. It is certainly possible that the lack of an effect of early life stressors among Met carriers might not similarly be apparent in the case of severe abuse or neglect. Thus, the present findings are limited in the scope of the early adverse events that were considered, and generalizations beyond this population, in which only quite mild abuse was reported, would be inappropriate. Finally, the number of participants that were assessed was moderate and precluded more detailed analyses that might have been important, including the contribution of cultural factors and gender, both of which can interact with early life events as well as genotype in predicting later outcomes. In this regard, previous reports revealed sex differences in coping style [29], [64], just as females exhibit higher levels of depression. In the current investigation, we assessed the potential interaction between the Val66Met polymorphism and sex in relation to coping. Although sex differences were not evident with respect to problem-focused coping, both emotion-focused and avoidant coping was used more in females than in males, but genotype and sex did not interact to predict either coping style. However, the limited number of participants in the present study may have obfuscated effects that might otherwise have occurred. This is especially the case as the relationship between sex and neglect may have varied with the age at which these experiences occurred, but the small N in the present study did not permit a meaningful analysis of this variable’s contribution.

Together, the results of the current investigation indicate that the Val66Met BDNF polymorphism, and by association neurotrophic support, interacts with early life stress to predict depressive symptoms. The BDNF polymorphism moderated the relationship between childhood neglect and subsequent depressive symptoms, such that this link was predominantly evident in the presence of Val/Val alleles. Interestingly, the Met allele did not represent a vulnerability factor in the relation between early life neglect and later depressive features. To the contrary, it appeared as if the Met allele served to limit the relationship between these factors. It was also observed that greater use of emotion-focused coping style was associated with the Met polymorphism. However, once more, it was only among the Val/Val individuals in which neglect was associated with increased emotion-focused coping styles as well as diminished perceived control, both of which predicted greater depression scores. The data of the present investigation are correlational, thus precluding causal statements regarding the direction of effects. Nevertheless, the data are consistent with the view that neglect affects depression through actions on coping styles and perceived coping flexibility, and that these relations are moderated by the presence of gene polymorphisms associated with neurotrophic support.
